# Short birth interval and its associated factors among multiparous women in Mieso agro-pastoralist district, Eastern Ethiopia: A community-based cross-sectional study

**DOI:** 10.3389/fgwh.2022.801394

**Published:** 2022-09-07

**Authors:** Musa Mohammed Wakeyo, Jemal Yusuf Kebira, Nega Assefa, Merga Dheresa

**Affiliations:** ^1^Chiro General Hospital, Oromia Regional Health Bureau, Addis Ababa, Ethiopia; ^2^School of Public Health, College of Health and Medical Sciences, Haramaya University, Harar, Ethiopia; ^3^School of Nursing and Midwifery, College of Health and Medical Sciences, Haramaya University, Harar, Ethiopia

**Keywords:** short birth interval, agro-pastoralist communities, multiparous, Oromia, Ethiopia

## Abstract

**Background:**

Recently, the concern with birth interval has acquired importance in public health and family planning because of its implication for fertility, maternal, and child health. A short birth interval is associated with adverse perinatal, maternal, and infant outcomes. Moreover, too short birth interval lead to high fertility, which in turn contributes to accelerated population growth and undermines development efforts. This study aimed to investigate the prevalence of short birth interval and its associated factors among multiparous women in the Mieso agro-pastoralist district, Oromia region, Eastern Ethiopia.

**Methods:**

A community-based cross-sectional study was conducted from 1 to 30 March 2020. The multistage sampling technique was used to select 490 multiparous women. Data were collected by face-to-face interviewer-administered structured questionnaires. Bivariate and multivariable logistic regression analyses were executed. Model fitness and multicollinearity were checked. Statistically significant associations of outcome and independent variables were declared at a *P*-value of < 0.05.

**Results:**

The prevalence of short birth interval was 56% (95% CI: 51.4–60.5) in the study area. Being married under 18 years (AOR = 3.78, 95% CI: 1.97–7.25), having formal education (AOR = 0.23, 95% CI: 0.11–0.47), having a husband with formal education (AOR = 0.46, 95% CI: 0.22–0.99), having awareness about optimum birth interval (AOR = 0.47, 95% CI: 0.24–0.91), having female index child (AOR = 1.78, 95% CI: 1.07–3.84), death of the index child (AOR = 0.34, 95% CI: 0.12–0.92), breastfeeding of the index child <24 months (AOR = 2.6, 95% CI: 1.53–4.41), use of modern contraceptive (AOR = 2.09, 95% CI: 1.12–3.89), and decision-making by a husband alone when to have a child (AOR = 3.86, 95% CI: 2.06–7.21) were significantly associated with short birth interval at a *P*-value <0.05.

**Conclusion:**

The overall prevalence of short birth interval among the study participants was high, as more than half of the women had practiced short birth interval, indicating that the majority of the mother and children in the study area are still at high risk of mortality and morbidity associated with short birth interval. Thus, the current findings suggest that interventions that involve the provision of contraceptives and information on its benefit at points need to be adopted to reach the national and global target of maternal and child mortality reduction attributed to short birth interval.

## Introduction

Inter-birth interval is defined as the time elapsed between two consecutive births (1). It has received special attention in public health and family planning because of its implication for fertility, maternal, and child health. The World Health Organization (WHO) recommends at least 33 months of an inter-birth interval between two consecutive live births. Inter-birth interval of <33 months is considered a short birth interval, and between 36 and 59 months is considered the optimum birth interval (2). Worldwide, although the practice of birth intervals differs, more than half (54%) of women in developing countries have had short birth interval (3). Women in sub-Saharan African countries also often have short birth interval (4, 5). In Ethiopia, the prevalence of short birth interval has been reported to be high, ranging from 21.7% (national estimate) (6) to 59.9% (district level) (7).

Empirical evidence from various studies identified that a relationship prevails between birth interval and maternal, infant, and child health (8–11). short birth interval increase the adverse health consequences for newborn, child, and maternal health. Evidence shows that a short birth interval has been associated with an increased risk of adverse perinatal health outcomes such as preterm birth, low birth weight (11–13), neurodevelopmental delay (14, 15), and perinatal or neonatal mortality (16, 17). It has also been associated with adverse maternal health outcomes such as maternal death, anemia, and gestational diabetes (18, 19). Beyond the maternal and child health implications, too short birth interval lead to high fertility, which in turn accelerates population growth and undermines development efforts by restraining women's contribution to economic growth (20).

Ethiopia has made substantial changes in reducing maternal and neonatal mortality over the last two decades (21). As a strategy to promote maternal and child health, the Ethiopian Federal Ministry of Health recommends spacing childbirth at intervals of 3–5 years to reduce maternal, perinatal, and infant mortality by optimizing the fertility rate in the country (22). However, over half of the non-first birth remains to occur within 3 years of their prior birth (6). In contrast, although, the county has shown remarkable changes in fertility reduction over the past two decades (23), fertility remained high, especially in the pastoralist area where fertility is too high, with a total fertility rate of above six per woman (6, 24, 25). Consistent with high fertility, the majority of births in Ethiopia (62%) fall into one of the high-risk categories. A short birth interval remains one of the main single risk factors that attribute to many risk full births, particularly common for women in the pastoralist communities (25).

Some previous studies done in Ethiopia have documented the factors shown to have been associated with short birth interval (26–29). However, those studies have been limited to other areas, with no consideration of women in disadvantaged pastoral communities. Therefore, it is essential to generate evidence concerning short birth interval and determine the factors shown to have increased the risk among disadvantaged women in pastoral communities of Ethiopia. Moreover, to the best of the author's knowledge, none of the studies have been conducted to assess this public health and development priority theme in the study area. Thus, this study aimed to determine the prevalence of short birth interval and their associated factors among multiparous women in the Mieso agro-pastoralist district, eastern Ethiopia. The findings of this study can help policymakers and public health planners target specific groups of women in continued efforts of reducing the burden of short birth interval on maternal and child health in the study area.

## Methods and materials

### Study design, setting, and period

A community-based cross-sectional study was conducted in the Mieso agro-pastoralist district, Oromia region, Eastern Ethiopia, from 1 to 30 March 2020. The district is located in Eastern Ethiopia, 300 km far from Addis Ababa, the capital city of Ethiopia. The total population (2019 projection based on the 2007 Census, CSA) of the district was 139,186. The district is categorized as a semi-pastoral district. Women of reproductive age comprise about 22% of the total population of the district (30). For administrative purposes, the district is subdivided into 33 kebeles (the smallest administrative unit in Ethiopia).

### Population, inclusion, and exclusion criteria

The source population was all women of childbearing age, and the study subjects encompassed 490 multiparous women in the study. Inclusion criteria include having a minimum of two successive live births and at least the last birth was within the last 2 years before data collection, whereas women were excluded if they had any history of stillbirths and abortion in between the last two successive live births. In addition, women who had health problems that hinder them not to respond to the interview were excluded.

### Sample size determination and sampling technique

The sample size for this study was calculated by considering the following assumptions with a single population proportion formula [*n* = (Z α/2)^2^ P (1–P)/d^2^]; by taking a 24.6% proportion of short birth interval among women from a previous similar study conducted in rural Bangladesh (31), 95% confidence level, 4% tolerable margin of error, and 10% expected non-response rate. The final sample size was 490.

**Figure 1:**
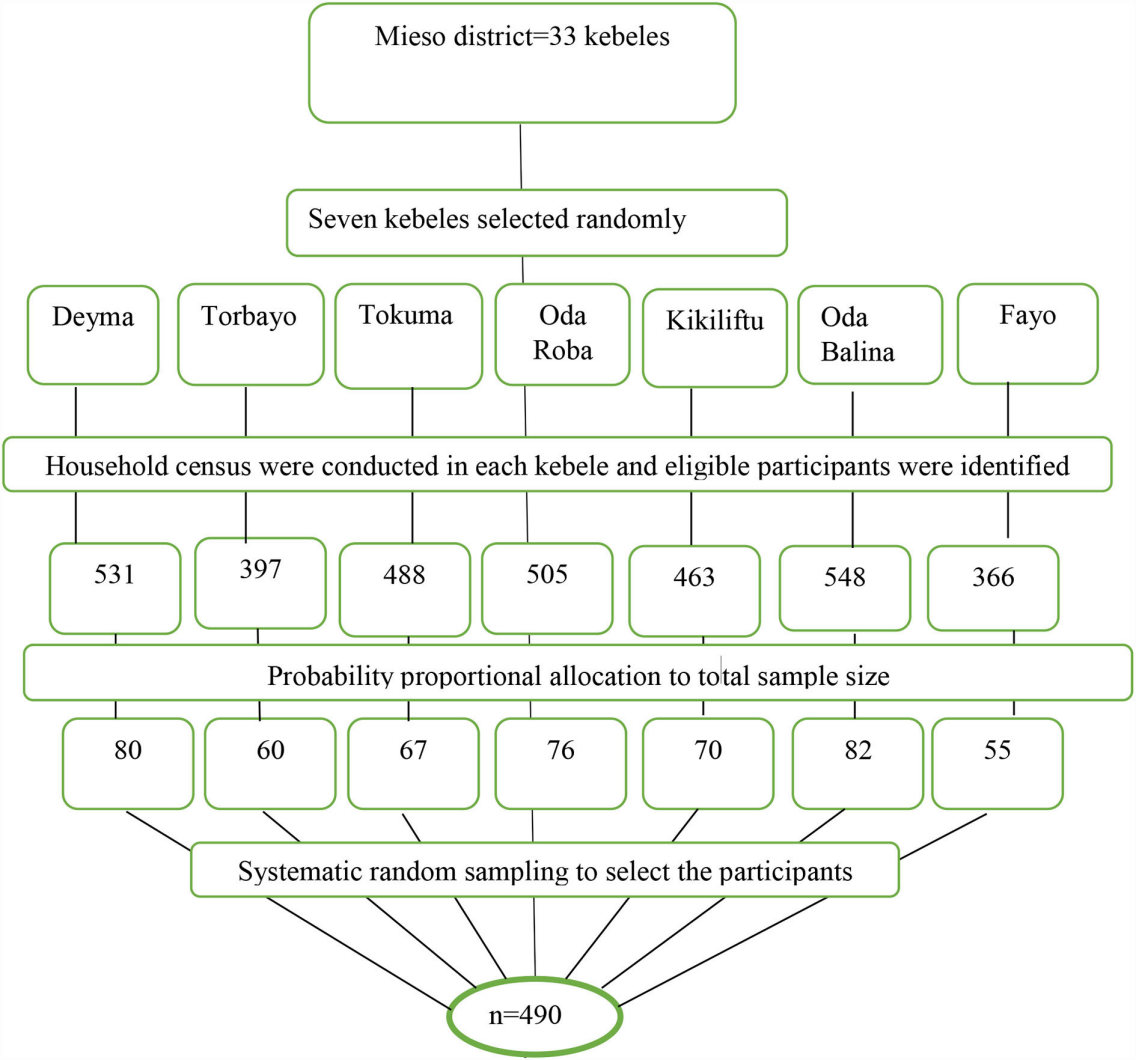
A diagrammatic flowchart of sampling technique for short birth interval and its associated factors among multiparous women in Mieso agro-pastoralist district, Eastern Ethiopia, 2020.

A multistage sampling method was used to select the representative study participants. First, seven kebeles from 33 kebeles in the district were selected randomly. Second, household census and coding were carried out in each randomly selected kebele by health extension workers to obtain a sampling frame (list of eligible participants) before the actual data collection commencement. Accordingly, a total of 3,258 households with eligible participants were identified. The probability to proportional sampling was used to allocate the total sample size proportional to the size of study subjects in each kebele. Finally, a household with eligible study participants was selected using a systematic sampling technique. Household sampling interval (K) was calculated by dividing the total number of households with eligible participants by the total number of study participants; K = N/n, 3,528/490 = 7.2. Thus, the sampling interval (K) became 7, and the first household with an eligible participant was selected using the lottery method (Figure 1).

### Data collection tools and techniques

Data were collected using a face-to-face interviewer-administered structured questionnaire. The interview consisted of the sociodemographic profile of the mother, reproductive history, and awareness about birth interval, breastfeeding, and modern contraceptive-related factors. The questionnaire was designed by reviewing related literature (5, 7, 26, 32) and adapted to the local situation. A total of 10 personnel participated, seven health extension workers and three midwifery professionals, in the data collection and supervision processes, respectively. Following recruitment, information has been provided about the data collection instruments and revisits for closed houses on the second day and consideration for replacement if absent on the second day. The birth date of the last and index child was determined by either the child's immunization card or the mother's recall.

### Data quality assurance

Data quality assurance mechanisms were carefully developed and implemented at various stages of the study. Initially, the questionnaire was prepared in English and then translated into the local language (Afan Oromo). It was pretested on 5% of the sample size in Asebot Kebele (the nearest non-studied kebele) and checked for consistency as well as appropriateness for the study participants 2 weeks before actual data collection commencement. After the pretest, some modifications were made to the initially prepared questionnaire. The training was given to data collectors and supervisors on the study procedures, data collection steps, and supervision activities for 2 days. Double data entry was done by two data clerks and cross-checked to ensure consistency. Collected data were checked for completeness daily by supervisors.

### Measurable outcome variable

In this study, the birth interval was determined by asking the women to present the birth certificates of the index (previous) and the last (recent) child. Alternatively, if a birth certificate was not available, immunization cards were used. In case both birth certificates and immunization cards were not available, the mother's memory was used to estimate the birth date of the index and the last birth. Next, the birth interval was determined by subtracting the time gap between the two birth dates and expressed in months. To be consistent with the World Health Organization's recommendation (2) and previous similar studies (26, 27, 32), the calculated birth interval was classified as <33 and ≥ 33 months. Then, if the calculated birth interval was <33 months, women were assumed to be practicing short birth interval. Finally, the outcome variable (short birth interval) was dichotomized and an overall binary variable was created with a value of Yes and No, where Yes indicated short birth interval, and No indicated no short birth interval.

### Operational definitions

#### Birth interval

The period between two successive live births, from birth date to birth date.

#### Short birth interval

In this study, a short birth interval was operationalized as an “inter-birth interval of <33 months between two consecutive live births.”

#### Optimum birth interval

A birth interval of 33 months and above between the birth of the index child and the immediately preceding live birth.

#### Index child

A child who preceded the last child.

### Data processing and analysis

Collected data were coded, entered, and cleaned using EpiData version 3.1 (EpiData Association, Odense, Denmark) and analyzed using Statistical Package for Social Sciences, SPSS version 23 (IBM Corp., Armonk, NY, U.S.A). Data cleaning was performed to identify outliers or inconsistencies, errors, and missing values. Both descriptive and analytical statistics were executed. Descriptive statistics such as mean (standard deviation) and median for continuous data as well as frequency and percentage for categorical data were used to describe the participant's characteristics. Both binary and multivariable logistic regression models were used to determine the association between the independent and the outcome variables. The model fitness was checked using the Hosmer-Lemeshow test (P = 0.632). Multicollinearity was also checked using variance inflation factor (VIF) and tolerance to see the correlation between independent variables. First, bivariate analysis was done to determine the association between each independent and the outcome variables as well as variables that were candidates for multivariable analysis. The results of the bivariate analysis were expressed as crude odds ratios (COR) with 95% confidence intervals (CI). All factors that had an association with the outcome variable at a *p*-value of 0.25 or less in bivariate analysis were entered into the multivariable model. Finally, statistical significance was considered at a *p*-value < 0.05 using an adjusted odds ratio (AOR) with a 95% CI.

## Results

### Sociodemographic characteristics of the study participants

A total of 484 mothers participated in the study, yielding a response rate of 98.8%. The mean age of women at the time of the interview was 31.2 (SD = ± 6.7), ranging from 20 to 49 years. The median age at first marriage for the study participants was 18 years, with the minimum and maximum age of marriage being 12 and 33 years, respectively. The majority of the participants, 449 (92.8%), were Muslims, and 445 (91.9%) were from Oromo ethnic groups. There were 267 (55.2%) women who were housewives. A majority, 442 (91.3%), of the study participants were living with their husbands. Of the participants, 282 (58.3%) had formal education, whereas 188 (38.8%) of their husbands had no formal education. One-third of the study participants, 164 (33.9%), had earned an average monthly income > 5,000 Ethiopian Birr (Table 1).

**Table 1:** Sociodemographic characteristics of multiparous women in Mieso agro-pastoralist district, Oromia region, Eastern Ethiopia, 2020 (*n* = 484).

**Variables**	**Categories**	**Frequency**	**Percentages**
Age group (years)	20–29	219	45.2
	30–39	208	43.0
	≥40	57	11.8
Religion	Muslim	449	92.8
	Non-Muslim	35	7.8
Age at first marriage (years)	<18	183	37.8
	≥18	301	62.2
Ethnicity	Oromo	445	91.9
	Somali	10	2.1
	Amhara	19	3.9
	Others*	10	2.1
Current marital status	With spouse/in marriage	442	91.3
	Not spouse/not in marriage	42	8.7
Education status	No formal education	202	41.7
	Formal education	282	58.3
Occupation status	Housewife	267	55.2
	Employee (GO/NGO)	89	18.4
	Others**	128	26.4
Husband's education	No formal education	188	38.8
	Formal education	296	61.2
Husband's occupation	Farmers	266	55
	Employee (GO/NGO)	112	23.1
	Others***	106	21.9
Average monthly income	<2,000 ETB	90	18.6
	2,000–3,500 ETB	158	32.6
	3,501–5,000 ETB	72	14.9
	>5,000 ETB	164	33.9

ETB, Ethiopian Birr; GO, governmental organization; NGO, non-governmental organization; others^*^: Afar, Gurage, Argoba; others^**^: merchant, pastoralist; others^***^: Pastoralist, daily workers, merchant.

### Reproductive history, awareness about birth interval, and child status characteristics of the study participants

The median duration of the birth interval between the index and the last child was 31 months. More than two-thirds, 336 (69.4%), of the study participants heard about the optimum birth interval between two successive births. For both mother and child, 357 (76.4%) participants responded that an optimum birth interval has health benefits. Nearly one-third, 166 (34.1) of the participants had 3 or 4 live births. More than half of the participants, 254 (52.5%), delivered their index child at home, and 80 (16.5%) women reported the death of the index child before the conception of the last child. The majority, 465 (96.1%), delivered the index child through the vagina. Moreover, 289 (59.97%) women showed the intention to have more children (Table 2).

**Table 2:** Reproductive history, awareness about birth interval and child status characteristics among multiparous women in Mieso agro-pastoralist district, Oromia region, Eastern Ethiopia, 2020 (*n* = 484).

**Characteristics**	**Categories**	**Frequency**	**Percentages**
Heard about the optimum birth interval between two successive live birth	Yes	336	69.4
	No	148	30.6
Optimum birth interval has health benefits for the mother and the child	Yes	370	76.4
	No	5	1.0
	I don't know	109	22.5
Short birth interval has health risks for the mother and the child	Yes	351	72.5
	No	12	2.5
	I don't know	121	25
Number of live births	2	135	27.9
	3–4	163	33.7
	≥5	186	38.4
Multiple births in the index child	Yes	26	5.4
	No	458	94.6
Place of delivery of the index child	Health facility	230	47.5
	Home	254	52.5
Mode of delivery of index child	Cesarean section	19	3.9
	Vagina	465	96.1
Survival status of the index child before conception of the last child	Alive	404	83.5
	Died	80	16.5
Sex of the index child	Male	270	55.8
	Female	214	44.2
Intention to have more children after the last child	Yes	289	59.7
	No	195	40.3
Preferred birth intervals (months)	<33	208	43
	33–59	194	40.1
	>59	82	16.9
Decision making status when to have the next child	Together	245	50.6
	Husband alone	204	42.1
	Self	35	7.2

### Breastfeeding and modern contraceptive practices among the study participants

The majority, 444 (91.7%), of the women in the study area have breastfed their index child. Among those who had breastfed their index child, 245 (55.2%) participants had breastfed for <24 months. Regarding the study participant's awareness of modern contraceptives, 447 (92.4%) participants had heard about modern contraceptives. nevertheless, only 40.1% of them have reported utilization of modern contraceptives before the conception of their last child. A majority, 178 (91.8%), of the study participants had utilized modern contraceptives for spacing, whereas 16 (8.2%) had used them for limiting births. In addition, 183 (37.8%) of the participants utilized one of the modern contraceptive methods at the time of the interview. The remaining study participants did not utilize modern contraceptives at the time of the interview for different reasons they have mentioned (Table 3).

**Table 3:** Breastfeeding and modern contraception practice among multiparous women in Mieso agro-pastoralist district, Oromia region, Eastern Ethiopia, 2020 (*n* = 484).

**Characteristics**	**Categories**	**Frequency**	**Percentages**
Breastfeed the index child	Yes	444	91.7
	No	40	8.3
Duration of breastfeeding in the index child (*n* = 444) (months)	<24	245	55.2
	≥24	199	44.8
Women's preference on the duration of breastfeeding (months)	<24	138	28.5
	≥24	346	71.5
Awareness of modern contraceptives	Yes	447	92.4
	No	37	7.6
Modern contraceptives used before the conception of the last child	Yes	194	40.1
	No	290	59.9
Reason for using modern contraceptives (*n* = 194)	Birth spacing	178	91.8
	Limiting of birth	16	8.2
Current use of modern contraceptives	Yes	183	37.8
	No	301	62.2
Place where women got modern contraceptives (*n* = 183)	Health post	55	30.1
	Health center	102	55.7
	Hospital	21	11.5
	Private clinic	5	2.7
Reason for not using contraception (*n* = 301)	Desire more child	71	23.6
	Religious reason	45	15.0
	Health problems	27	9.0
	Lack of autonomy	64	21.3
	Others*	94	31.2

Others^*^: lack of the service nearby, lack of information, cultural barriers.

### The prevalence and factors associated with short birth interval among the study participants

Of the study participants, 271 (56%) (95% CI: 51.4–60.5) had practiced short birth interval. The median duration of the birth interval was 31 months.

Initially, the sociodemographic characteristics of respondents, reproductive history, and birth interval awareness, as well as breastfeeding and modern contraceptive practice, were analyzed with bivariate logistic regression to determine factors associated with short birth interval. Finally, variables with a *p*-value of <0.25 in bivariate logistic regression were fitted into multivariate logistic regression. Accordingly, being married under 18 years (AOR = 3.78, 95% CI: 1.97–7.25), having formal education (AOR = 0.23, 95% CI: 0.11–0.47), having had a husband with formal education (AOR = 0.46, 95% CI: 0.22–0.99), having awareness about optimum birth interval (AOR = 0.47, 95% CI: 0.24–0.91), having female index child (AOR = 1.78, 95% CI: 1.07–3.84), death of the index child (AOR = 0.34, 95% CI: 0.12–0.92), breastfeeding of the index child <24 months (AOR = 2.6, 95% CI: 1.53–4.41), use of modern contraceptive before conception of the last child (AOR = 2.09, 95% CI: 1.12–3.89), and decision-making by the husband alone when to have a child (AOR = 3.86, 95% CI: 2.06–7.21) were significantly associated with a short birth interval at a *P*-value <0.05 (Table 4).

**Table 4:** Factors associated with short birth interval among multiparous women in Mieso agro-pastoralist district, Oromia region, Eastern Ethiopia, 2020 (*n* = 484).

**Variables**	**Short birth interval**				
	**Yes**	**No**	**COR (95% CI)**	**AOR (95% CI)**	***P*-Value**
	***N* (%)**	***N* (%)**			
Current marital status					
Living with husband	254 (57.1)	191 (42.9)	1	1	
Not living with husband	17 (43.6)	22 (56.4)	1.72 (0.89, 3.33)	1.94 (0.64, 5.86)	0.239
Age at first marriage					
≥18	191 (63.0)	112 (37.0)	1	1	
<18	80 (44.2)	101 (55.8)	2.15 (1.48, 3.13)	3.78 (1.97, 7.25)^*^	0.001
Religion					
Non-Muslim	16 (45.7)	19 (54.3)	1	1	
Muslim	255 (56.8)	194 (43.2)	1.56 (0.72, 2.87)	1.49 (0.82, 3.15)	0.997
Educational status					
No formal education	88 (43.6)	114 (56.4)	1	1	
Formal education	183 (64.9)	99 (35.1)	0.42 (0.29, 0.61)	0.23 (0.11, 0.47)^*^	0.001
Husband's education					
No formal education	86 (45.7)	102 (54.3)	1	1	
Formal education	185 (62.5)	111 (37.5)	0.51 (0.35, 0.74)	0.46 (0.22, 0.99)^*^	0.047
Husband's occupation					
Farmer	155 (58.3)	111 (41.7)	1	1	
Employee (GO/NGO)	44 (39.3)	68 (60.7)	2.2 (1.38, 3.39)	1.08 (0.44, 2.65)	0.867
Others	72 (67.9)	34 (32.1)	0.66 (0.41, 1.1)	0.48 (0.22, 1.05)	0.065
Optimum birth interval awareness					
Yes	170 (50.6)	166 (49.9)	0.48 (0.31, 0.72)	0.47 (0.24, 0.91)^*^	0.012
No	101 (68.2)	47 (31.8)	1	1	
Total live births					
2	69 (51.1)	66 (48.9)	1	1	
3-4	97 (59.5)	66 (40.5)	0.71 (0.45, 1.13)	0.76 (0.36, 1.61)	0.475
≥5	105 (56.5)	81 (43.5)	0.81 (0.52, 1.26)	0.99 (0.46, 2.10)	0.968
Sex of index child					
Male	171 (64.0)	96 (36.0)	1	1	
Female	100 (46.1)	117 (53.9)	2.08 (1.45, 3.0)	1.78 (1.07, 3.84)^*^	0.001
Survival status of the index child					
Alive	207 (51.2)	197 (48.9)	1	1	
Died	64 (80.0)	16 (20.0)	0.26 (0.15, 0.5)	0.34 (0.12, 0.92)^*^	0.034
Place of delivery of index child					
Health institution	138 (60.0)	92 (40.0)	1	1	
Home	133 (52.4)	121 (47.6)	1.36 (0.95, 1.96)	1.1 (0.57, 1.99)	0.856
Mode of delivery in index child					
Caesarean section	14 (73.7)	5 (26.3)	2.27 (0.8, 6.39)	2.3 (0.53, 10.0)	0.268
Vagina	257 (55.3)	208 (44.7)	1	1	
Multiple births in index child					
Yes	21 (80.8)	5 (19.2)	3.49 (1.3, 9.43)	3.71 (0.73, 9.43)	0.115
No	250 (54.8)	208 (45.4)	1	1	
Duration of breastfeeding the index child					
<24 months	206 (84.1)	39 (15.9)	3.54 (1.61, 4.25)	2.6 (1.53, 4.41)^*^	0.001
≥24 months	26 (13.1)	174 (86.9)	1	1	
Used modern contraceptive before a last child					
Yes	122 (62.9)	72 (37.1)	1.6 (1.11, 2.32)	2.09 (1.12, 3.89)^*^	0.043
No	149 (51.4)	141 (48.6)	1	1	
Decision-making status when to have a child					
Husband alone	163 (66.5)	82 (33.5)	2.42 (1.65, 3.55)	3.86 (2.06, 7.21)^*^	0.001
Self	16 (45.7)	19 (54.3)	2.36 (1.15, 4.83)	3.78 (0.97, 14.76)	0.055
Decide together	92 (45.1)	112 (54.9)	1	1	

* Significant at *P*-Value <0.05 for AOR; ETB, Ethiopian Birr; CI, Confidence Interval; COR, Crude Odd Ratio; AOR, Adjusted Odd Ratio; others: Pastoralist, daily workers, merchant.

## Discussion

This study aimed to determine the prevalence of short birth interval and their associated factors among multiparous women in the Mieso agro-pastoralist district, eastern Ethiopia. The overall prevalence of short birth interval among the study participants was high in the study area. Only a few over one-third of participants had used modern contraceptives. A significant association was observed between short birth interval and certain sociodemographic, child, and reproductive health characteristics of the participants.

Findings of this study revealed that more than half (56%) of women in the study area were practicing short birth interval, which is consistent with the study conducted in Uganda (52.4%) (5), Southwest Ethiopia (59.9%) (7), Kasala, Eastern Sudan (60.6%) (33), and Southern Ethiopia (57%) (34). However, the result of this study is higher than the findings of the study conducted in rural Bangladesh (24.6%) (31), Nepal (23%) (35), Brazil (17.1%) (36), Northern Ethiopia (23.3%) (26), and Arsi Zone, Southeast Ethiopia (17.3%) (29). This discrepancy could be explained by the difference in the measurement of short birth interval, study settings, and interventions made across countries. Some of the previous studies used cutoff point measurement for short birth interval of 24 months (35, 36), while this study used 33 months. In addition, the variation could be due to the difference in the contextual regions in which women live. The nomadic feature of women in pastoralist areas hinders access to modern contraceptive services (37, 38), which could attribute to a high proportion of short birth interval among the women in pastoralist areas.

This study found an association between age at first marriage and short birth interval practice. Women who had married at an age <18 years were more likely to practice short birth interval compared to those who had married at age 18 years or above. This finding is in line with the study done in Jordan (39) and Southwest Ethiopia (7), which reported early marriage before the age of 18 years increased the likelihood of experiencing short birth interval. The reason might be because younger women are more likely to have children for a variety of reasons such as greater fecundity and being early in the family-building process (40).

In this study, maternal education was significantly associated with short birth interval, indicating that the odds of having short birth interval were lower among mothers who had attended formal education as compared to their counterparts who had no formal education. This finding is consistent with evidence from the studies conducted in Pakistan (41), Jordan (39), Arba Minch district, Ethiopia (28), and the pastoralist communities of Borena, South Ethiopia (32). This may be explained by the fact that educated mothers can have better health-seeking behaviors and decision-making skills in family planning service utilization.

Consistent with the findings of a study done in Arba Minch district, Ethiopia (28), and Dabat, Northwest Ethiopia (42), the findings of this study indicated that women whose husbands had attended formal education were less likely to have short birth interval compared to women whose husbands had no formal education. This might be because educated husbands possess knowledge about the health benefits of birth spacing, thus influencing their wives to seek family planning services and provide information about the importance of optimal birth spacing.

It has also been found that the sex of the index child was significantly associated with short birth interval in this study, in which mothers who had a female index child were more likely to have short birth interval compared to mothers who had a male index child. This finding is supported by the studies done in Babol, Iran (43), Nigeria (44), rural communities of Southern Ethiopia (32), Northern Ethiopia (45), and Dessie town, Ethiopia (27), which reported having a female sex index child increased the risk of short birth interval. This may be attributed to the reason that men are considered family head and guard in the pastoral community of Ethiopia (46), indicating mothers who had a female child in the previous birth may be eager to give birth until they get the desired number of male children who safeguard their cattle and families from enemies. In addition, it might be due to the prevailing culture of male preference among most pastoralist communities in Ethiopia (47), in which, if the preceding birth was a female child, a mother might be less likely to delay birth as desires to have a male child sooner.

The survival status of the index child was another factor associated with short birth interval in this study. Mothers whose index child was dead before conception of the last child were less likely to have short birth interval compared to those with a surviving index child. This finding is in contrast with the findings of the studies done in Uganda (48), Pakistan (49), Northwest Ethiopia (42), and Illubabor Zone, Southwest Ethiopia (50), which revealed that mothers whose index child was dead were more likely to have short birth interval than their counterparts. Even though there is no clear explanation for this contradiction, a hypothesis could be that mothers whose index child was dead included in this study might have an adverse maternal outcome that necessitates the mother to delay subsequent births.

Reproductive decision-making when giving birth was also associated with short birth interval in this study. Women whose husbands decided alone when to have a child were more likely to practice short birth interval than their counterparts. This finding is consistent with a study done in Uganda (5), which reported that women who were influenced by their husbands' decisions concerning when to have a child were more likely to have short birth interval. The possibility for this association may be that husbands' decisions might put a constraint on their wives not to use modern contraceptives, which in turn contributes to short birth interval.

Duration of breastfeeding was a significant factor associated with a short birth interval in this study. Those mothers who breastfed their index child for <24 months were more likely to have short birth interval compared to their counterparts. This concurs with the findings from studies done in Tigray, Ethiopia (26), and Borena, Southern Ethiopia (32). This might be because breastfeeding could delay postpartum amenorrhea and appears to have suppressed the ovulatory cycle (51), which in turn delays birth interval duration.

As found in this study, the odds of having short birth interval were higher among the mothers who had not used modern contraceptives before conception of the last child compared to those who had used it. This finding is supported by the studies conducted in Egypt (41), Ahvaz, Iran (52), Jordan (39), and Tigray, Ethiopia (26), which revealed that the odds of having short birth interval were higher among mothers who did not use modern contraceptives before getting pregnant with the last child than those who used. The purpose of the contraceptive method is either to limit or space births.

In conclusion, this study illustrated that the prevalence of short birth interval among the study participants was high, as more than half of the women had practiced short birth interval, indicating the majority of the mothers and children in the study area are still at high risk of mortality and morbidity associated with short birth interval. Thus, the current findings suggest that interventions that involve the provision of contraceptives and information on its benefit at points need to be adopted to reach the national and global target of maternal and child mortality reduction attributed to short birth interval. More importantly, ensuring gender equality needs to be a concern, as this would warrant the use of contraceptives and enhance reproductive health decision-making capacity among women. Furthermore, future qualitative studies are required to explore the social and cultural influences on the birth interval that are not addressed in this study.

This study has admitted a few limitations. First, this study might be exposed to recall bias since birth interval estimation was mainly based on the mother's memory. Second, being a cross-sectional study design, it does not establish cause-effect relationships between the study variables. Third, the study did not incorporate qualitative exploration of social and cultural influences on birth interval. Moreover, this study recruited a limited sample size, and hence the findings of this study may not be generalizable to women in other areas of Ethiopia.

## Data availability statement

The original contributions presented in the study are included in the article/supplementary material, further inquiries can be directed to the corresponding author/s.

## Ethics statement

The study was approved by the Institutional Health Research Ethics Review Committee, College of Health and Medical Sciences, Haramaya University, Ethiopia with approval number (Ref. No: IHRERC/179/2020). Written and verbal informed consent was obtained from all the study participants before the interview.

## Author contributions

MW, JK, NA, and MD made a significant contribution to the conception of the idea and design, participated in proposal development and data collection, and analyzed and interpreted the data. JK wrote the original draft of the manuscript. JK, NA, and MD reviewed and edited the manuscript for important intellectual content. All authors read and approved the final manuscript.

## Conflict of interest

The authors declare that the research was conducted in the absence of any commercial or financial relationships that could be construed as a potential conflict of interest.

## Publisher's note

All claims expressed in this article are solely those of the authors and do not necessarily represent those of their affiliated organizations, or those of the publisher, the editors and the reviewers. Any product that may be evaluated in this article, or claim that may be made by its manufacturer, is not guaranteed or endorsed by the publisher.
